# Neurochemical changes in different brain regions induced by PACAP - relations to neuroprotection

**DOI:** 10.1186/2193-1801-4-S1-L56

**Published:** 2015-06-12

**Authors:** Dora Reglodi, Gabor Maasz, Zsolt Pirger, Adam Rivnyak, Dorottya Balogh, Adel Jungling, Balazs Fulop, Laszlo Mark, Andrea Tamas

**Affiliations:** Department of Anatomy, MTA-PTE “Lendület” PACAP Research Team, University of Pecs, Pecs, Hungary; Centre for Ecological Research, HAS, Balaton Limnological Institute, Department of Experimental Zoology, Group of Chemical Ecology and Neurobiology, Tihany, Hungary

**Keywords:** PACAP, neuroprotection, proteomics

Pituitary adenylate cyclase activating polypeptide (PACAP) is a neuropeptide with diverse occurrence and functions. One of the most well-known effects of PACAP is its strong neuroprotective effect. In this presentation we give an insight into recently described neurochemical changes induced by PACAP or altered by PACAP the lack of it. In an invertebrate model for Parkinson’s disease we found that PACAP effectively counteracts the dopamine-decreasing effect of rotenone, a mitochondrial neurotoxin. Similarly, in a 6-hydroxydopamine-induced rat model of Parkinson’s disease, we found that PACAP effectively increases dopamine levels. Furthermore, our proteomics analysis shows that PACAP treatment also counteracts the 6-OHDA-induced decrease in PARK-7 protein, effective against oxidative stress. Studying the role of endogenous PACAP, we found that PACAP-deficient mice show higher susceptibility to toxic agents causing degeneration of the substantia nigra dopaminergic neurons. Using proteomic analysis we revealed that the expression of numerous proteins is altered in the mesencephalon and striatum of knockout mice. Among the altered proteins, several are involved in metabolic processes, energy homeostasis, and structural integrity. ATP-synthase and tubulin beta-2A were expressed more strongly in PACAP-knockout mice. In contrast, the expression of more peptides/proteins markedly decreased in knockout mice, like pyruvate kinase, fructose biphosphate aldolase-A, glutathione S-transferase, peptidyl propyl cis-trans isomerase-A, gamma enolase, beta-synuclein and aspartate amino transferase. The altered expression of these proteins might partially account for the decreased antioxidant, cytoprotective and detoxifying capacity of PACAP-deficient mice. The described changes may provide further explanation for the neuroprotective potency of PACAP.

